# Factors that influence participation in physical activity in Chinese teenagers: perspective of school educators and parents in respect of the social ecology model

**DOI:** 10.3389/fpsyg.2025.1430002

**Published:** 2025-02-20

**Authors:** Donglin Hu, Shi Zhou, Zachary J. Crowley-McHattan, Zhiyun Liu

**Affiliations:** ^1^Department of Physical Education, Nanjing Agricultural University, Nanjing, China; ^2^Faculty of Health, Southern Cross University, Lismore, NSW, Australia; ^3^School of Physical Education and Educational Science, Tianjin University of Sport, Tianjin, China

**Keywords:** physical activity, children, adolescents, social ecology model, the grounded theory

## Abstract

**Introduction:**

The objective of this study was to explore the viewpoints of parents, teachers, and administrators on the factors influencing adolescent physical activity in China.

**Methods:**

The study employed semi-structured interviews with school teachers, school principals, government officers, and parents. Twenty-five participants were recruited from Jiangsu Province, China, and completed the interview.

**Results:**

The data collected were analysed using grounded theory within the social ecology model framework. The analysis identified 49 concepts across 19 subcategories and five main categories.

**Discussion:**

The resulting theoretical model, constructed using grounded theory, integrated five main categories: individual factors, family environment, school environment, community environment, and policy. This model provides a foundational understanding of the multifaceted influencing factors of adolescent physical activity in China.

## 1 Introduction

Participation in adequate amount of moderate-to-vigorous physical activities (MVPA) has been shown to be able to promote growth and physical fitness and reduce the risk of being overweight and obese in youth (Bull et al., 2020). The World Health Organization (WHO) physical activity guidelines recommend that children and adolescents aged 6–17 years should engage in at least 60 min of moderate-to-vigorous physical activity (MVPA) per day, including vigorous physical exercise at least 3 days per week (Bull et al., 2020). However, the current adherence rate to these guidelines among Chinese school-aged children and adolescents stands at a mere 30% (National Health Commission and The Ministry of Education, 2020). Notably, some studies showed a gradual decline in physical activity levels as the children getting older (Hong et al., 2020; Piercy et al., 2018; World Health Organization, 2019).

An individual’s health behaviour is influenced by a number of factors, not only at the individual level but also by the external social and physical environment (Sallis et al., 2015). McLeroy et al. proposed a socio-ecological model of health behaviour (McLeroy et al., 1988), which is a comprehensive framework based on multiple theories and helps to analyse the influences at the individual, interpersonal, organisation, community, and policy levels on health behaviour. Although several previous studies have explored the factors influencing physical activity among adolescents in China, most of them have only focused on the middle and proximal levels of the social ecology model (individual, interpersonal and organisation levels) (Hu et al., 2021; Liu et al., 2021; Luo et al., 2021; Quan and Lu, 2020). At present, in the context of China, there is a notable absence of studies that thoroughly encompass all five levels of the social ecology model in their analysis. Additionally, while a few studies have indeed utilised the social ecology model as a theoretical underpinning and adopted qualitative methods to examine factors influencing adolescent physical activity (Corr et al., 2019; Martins et al., 2015), qualitative research in this domain, especially within the context of China, remains markedly scarce.

In 1967, Barney Glaser and Anselm Strauss introduced the grounded theory and methodological system in their monograph, The Discovery of Grounded Theory: Strategies for Qualitative Research (Glaser et al., 1968). The grounded theory is widely recognised as a rigorous and effective method in qualitative research (Oktay, 2012). The core of the method is the data collection and analysis process. The iterative process of comparative analysis and theoretical sampling ensures the reliability and validity of the resulting theory. Through repeated coding and constant comparison, researchers are able to gradually construct a theoretical model that closely reflects the reality of the studied population. This process not only ensures the scientific rigor of the results but also enhances the replicability and generalizability of the findings (Charmaz and Thornberg, 2021). The aims of this study, with a focus on the adult perspective, were to construct an initial conceptual model of factors influencing physical activity (PA) in China’s youth, and to provide a pool of items for scale development for future studies. A semi-structured interview was performed on school educators, government officers and parents. With the application of the grounded theory the interview transcripts were coded, generalised, refined and extrapolated to identify the influencing factors of PA participation, from which a conceptual model of the factors influencing children and adolescents’ physical activity was constructed.

## 2 Methods

### 2.1 Research design

This study used a semi-structured in-depth interview to collect and analyse qualitative data. In our study, we strategically concentrated on eliciting insights regarding adolescent behaviour from adults, specifically educators and parents, to capture a more encompassing perspective. Furthermore, the perspectives of adults are instrumental in delineating the various environmental and social factors that influence adolescent physical activity. This information is vital in formulating a robust intervention framework. Although this methodology primarily centres on adult viewpoints, it facilitates a thorough understanding of the multifaceted dynamics influencing adolescent physical activity, thereby enriching the depth of findings from our study. During the interviews, different participants’ attitudes, experiences and views about PA among young people were explored, the collected information was categorised, and the relationships between the identified factors were analysed (Mik-Meyer, 2020). The study used ‘theoretical sampling’, one of the core procedures of the grounded theory approach, as opposed to the ‘probability sampling’ approach used in quantitative research. The “theoretical sampling” is an iterative and ongoing process that integrates data collection, coding, and theory building (Glaser et al., 1968).

### 2.2 Ethics approval

This study obtained approval by the Ethics Committee of Tianjin University of Sport, China, and the Human Research Ethics Committee of Southern Cross University, Australia (Approval number: Tianjin University of Sport 2020/03, Southern Cross University 2021/109).

### 2.3 Participants

Sample size can vary in qualitative research (Baker and Edwards, 2017). According to Quinn Patton (2002), the sample size depends on the study’s purpose and aim, the quality of the data, and the available time and resources. Previous researchers have utilised sample sizes ranging from 10 to 12 participants for their qualitative studies on physical activity (Saligheh et al., 2016; Schmidt et al., 2016; Warehime et al., 2019; Yüksel and Schoville, 2020), and shown that data saturation can be achieved in as few as 10 interviews (Sugiyama et al., 2010). Data saturation is one method used to determine an adequate sample size in qualitative studies (Francis et al., 2010) that refers to the point when a researcher no longer obtains substantive new information. In the current study, the researcher planned to recruit participants until the point where data saturation occurred, with a minimum of 15 participants.

All study participants signed an informed consent form, and their participation was entirely voluntary without receiving any financial benefit. The researcher contacted school principals and teachers of three local junior to senior high schools (school year 7–12), and asked them to reach students’ parents who met the eligibility criteria. In addition, the researcher contacted the relevant government departments to assist the researcher in recruiting government officers who met the inclusion criteria.

#### 2.3.1 Inclusion criteria

- Parents: (a) living in Nanjing City (China) at the time of the study, (b) with a child attending a junior or senior high school (school year 7–12), and (c) willing to participate in the interview.
- School Teachers: (a) working in a public or private high school during the study period, (b) with more than 4 years of teaching experience in physical education, and (c) willing to participate in the interview.
- School Principals: (a) working in a public or private high school during the study period, (b) with more than 4 years of teaching experience, and (c) willing to participate in the interview.
- Government officers: (a) working in a government education or sports department (e.g., Jiangsu Provincial Education Department, Jiangsu Provincial Sports Bureau), (b) with more than 4 years of work experience in the department, and (c) willing to participate in the interview.

#### 2.3.2 Exclusion criteria

An individual who was (a) unable or unwilling to complete the interview after agreeing to participate, or (b) unable to schedule an interview at a mutually convenient time for both the participant and the researcher.

### 2.4 Data collection process

**Figure 1:**
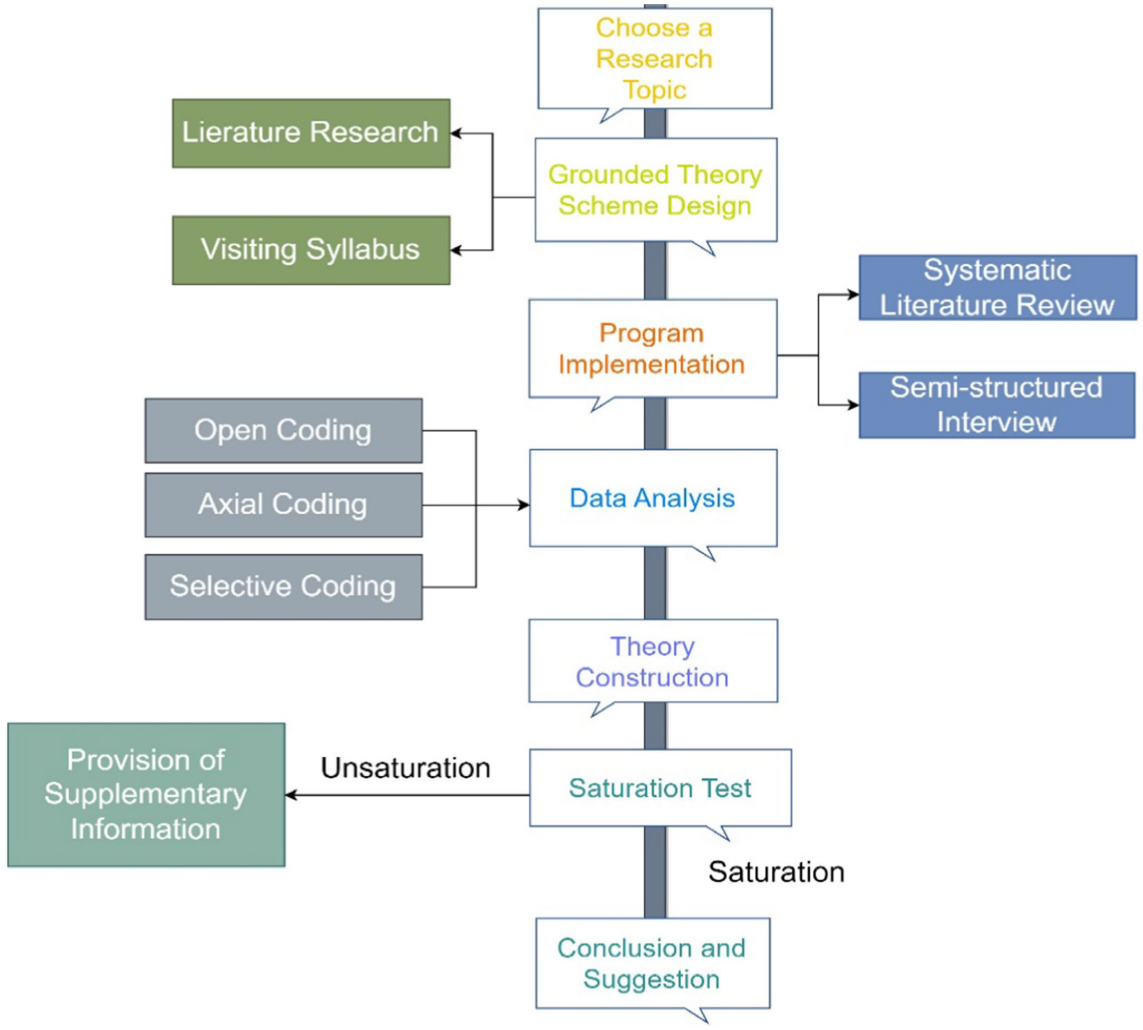
Schematic diagram of the grounded theory research process adapted from Oktay (2012).

This study aimed to develop a conceptual framework for exploring the factors influencing adolescent physical activity, using a socio-ecological model. The data collected during the exploration process was analysed using grounded theory. The analysis process involved comparative analysis, classification, and conceptualisation of the operational process, aiming to regroup the concepts according to logical relationships to develop a core theory. The theory sampling scheme consists of three methods: open coding analysis, axial coding analysis, and selective coding analysis. In the grounded theory coding process, conceptual categories were iteratively compared and relationships between concepts were established, as depicted in Figure 1.

In the coding and analysis process, we used the NVivo 12.0 software developed by Qualitative Solutions for Research (QSR) Ltd. as a supplementary analysis tool to conduct the semi-structured in-depth interviews with different groups of participants (parents, teachers, principals and government officers), using the social ecology model proposed by McLeroy et al. (1988) as a theoretical framework to examine the responses at the individual, interpersonal, organisation, community and policy levels, respectively.

First, the data were collected and recorded during the interview with the questions corresponding to the above-mentioned five levels. Secondly, the original interview data was imported into the database and encoded to refine the initial concepts and assigned to the categories and establish the initial nodes. Thirdly, axial coding was carried out to refine the main categories. Finally, the selective coding was carried out to discover the core categories and establish the links between the core categories, using the social ecology model as the theoretical framework to construct a theoretical model of the influencing factors of school students’ physical activity using the grounded theory.

### 2.5 The process of data recording

The data sources for this study were from the semi-structured in-depth interviews with parents, teachers, principals and government officers. The interview questions were derived from previous research literature (Hu et al., 2021; Langille and Rodgers, 2010; Webster and Suzuki, 2014). Interviews included explorations on the factors influencing current youth physical activity (both barriers and facilitators were included). Different questions were developed for the various roles (Table 1). Following the recommendations of the qualitative study (Mik-Meyer, 2020), we conducted an initial interview with an expert in the field of students’ physical health promotion (a professor from the Nanjing Normal University) before carrying out the formal interview. Subsequently, we revised the interview questions to improve clarity according to the feedback from the pilot interview.

**Table 1:** Semi-structured interview outline.

No.	Details
1	Find out basic information about the respondents, demographic information such as age, age of children, occupation, position, education, etc.
2	Can you tell us what factors (individual, family, school, community, policy) prevent young people from participating in physical activity?
3	What factors (individual, family, school, community, policy) would you like to discuss in promoting physical activity among young people?
4	Do you feel that children are getting enough physical activity and exercise now?
5	What do children do after school during the week? What do you do at home during holidays? (Parents)
6	Do you exercise regularly? Do you take your children with you when you exercise? (Parents)
7	Do you know about the physical education classes for children in school? Do you know what other sports activities are offered in addition to PE lessons? (Parents)
8	How many PE lessons does the school have a week? What are the contents of PE lessons? (Teachers, Principals)
9	Are there any other activities related to physical activity in school apart from PE lessons? (Teachers, Principals)
10	What is the rate of attainment of physical fitness standards for students? (Teachers, Principals)
11	Are there policies and regulations within the school that specifically promote physical activity for children? (Teachers, Principals)
12	What is the current level of physical activity of young people in Jiangsu province? (Government officers)
13	What efforts are currently being made in China or the Jiangsu Province to promote physical activity among young people? Is there any policy support? (Government officers)
14	Do you have any other ideas/suggestions? Please add

PE = physical education.

The researcher informed the interviewees of the purpose of the study and explained the measures to protect privacy of participants’ information before asking the participants to sign the informed consent form. The interviews were completed over 20–40 min, at a mutually agreed location face-to-face or via online video interview (due to the local COVID-19 pandemic-related restrictions). All interviews (online or face-to-face) were recorded using audio recording devices and notes were taken during the interview. Interviews were conducted from August to November 2021. The researcher converted the recordings and notes into text files at the end of the discussions and transferred the information into the Nvivo12 software. We employed professional transcribers skilled in handling various accents and speech patterns to ensure accurate transcription of interviews, which were recorded verbatim, including all spoken words and non-verbal cues. We conducted rigorous quality control by reviewing transcripts against audio recordings and, where possible, involved participants in reviewing their transcripts to validate accuracy. The final number of participants who completed the interview included nine parents, seven teachers, four principals and five government officers, with a total of 25.

To ensure the rigor and reliability of the findings, both internal and external validation procedures were applied. Internal validation was conducted through the constant comparison method during the data analysis process, ensuring that emerging themes were grounded in the data. External validation was performed by inviting independent experts to review the coding process and the final conceptual framework. These validation procedures were implemented at different stages of the research, including during the data analysis and after the initial conceptual model was developed.

In the coding and analysis process, we used NVivo 12.0 as a supplementary tool, employing the social ecology model by McLeroy et al. (1988) to analyse semi-structured interviews with parents, teachers, principals, and government officers at individual, interpersonal, organisational, community, and policy levels. First, data was collected and coded based on these five levels. For example, a teacher’s comment, “Over half of the students actively participated in the PE Cultural Festival,” was coded as “Sports atmosphere,” categorized under “School atmosphere.” Similarly, a parent’s response, “We encourage our child to stick with exercise,” was coded as “parental support” under “family support.” Axial coding was then used to refine categories like “family environment “and “school environment,” and selective coding followed to establish core categories and their relationships, forming a theoretical model of the factors influencing students’ physical activity.

## 3 Results

### 3.1 Demographic information

#### 3.1.1 Parents interviewed

The researcher invited a total of 15 parents, and 10 parents of students were willing to be interviewed. Nine parents (four fathers and five mothers) eventually completed the interviews at the agreed interview time and venue. Table 2 shows the socio-demographic characteristics of the parents interviewed.

**Table 2:** Socio-demographic characteristics of interviewed parents.

No.	Gender	Age	Occupation	No. of children	Children’s age/grade	Sex of child	School Type	Highest degree
P1	Female	43	Company clerk	1	18/Grade 12	Boy	Public	Bachelor’s degree
P2	Female	45	Self-employed	1	15/Grade 9	Girl	Public	Bachelor’s degree
P3^a^	Male	41	Company clerk	2	16/Grade 10	Girl	Public	High school qualification
P4	Female	42	accountant	1	13/Grade 7	Girl	Private	Master’s degree
P5^b^	Male	45	Bank Management	2	17/Grade 11	Boy	Public	Bachelor’s degree
P6	Male	48	Self-employed	1	17/Grade 10	Boy	Public	Bachelor’s degree
P7	Female	38	Company clerk	1	16/Grade 9	Girl	Private	Tertiary qualifications
P8^c^	Male	50	Self-employed	2	17/Grade 11	Boy	Public	High school qualification
P9^d^	Female	40	Researcher	1	14/Grade 8	Boy	Public	Master’s degree

P = Parents, a = having twin girls, both attending the same senior high school in different classes in the same grade; b = having an older child (boy) attending a senior high school; c = a younger child (boy) attending a middle high school; d = interview notes used for theoretical saturation test.

#### 3.1.2 Teachers and principals interviewed

A total of nine teachers and five principals were invited by the researcher, and seven teachers and four principals eventually completed the interviews. Table 3 shows the socio-demographic characteristics of the teachers and principals interviewed.

**Table 3:** Socio-demographic characteristics of interviewed teachers and principals.

No.	Gender	Age	Years of teaching	Discipline	Highest degree	Type of school	Comment
T1	Male	31	7	Physical Education	Bachelor’s degree	Public Junior and senior high school	Teacher
T2	Male	37	12	Physical Education	Master’s degree	Private Junior and senior high school	Teacher
T3	Male	55	35	Physical Education	Bachelor’s degree	Public Junior and senior high school	Director
T4	Male	38	15	Physical Education	Master’s degree	Private Primary, junior and senior high school	Vice director
T5	Male	45	22	Physical Education	Bachelor’s degree	Public Senior high school	Director
T6	Male	40	18	Physical Education	Master’s degree	Public Junior and senior high school	Headteacher
T7^a^	Male	38	14	Physical Education	Master’s degree	Public Junior and senior high school	Director
H1	Male	51	27	Physical Education	Master’s degree	Public Junior and senior high school	Vice-president
H2	Male	48	26	Physical Education	Master’s degree	Public Primary and junior high school	Vice-president
H3	Female	46	24	Chinese Language	Master’s degree	Public Junior and senior high school	Vice-president
H4^b^	Female	50	30	Politics	Master’s degree	Public Senior high school	Vice-president

T = Teachers, H = Principals, a = Interview transcripts for theoretical saturation test, b = Interview transcripts for theoretical saturation test.

#### 3.1.3 Government officers interviewed

The researcher invited a total of eight government officers and five eventually completed the interview. Table 4 shows the socio-demographic characteristics of the government officers interviewed.

**Table 4:** Socio-demographic characteristics of interviewed government officers.

No.	Gender	Age	Department	Position	Responsible area	Highest education
G1	Female	54	Jiangsu Institute of Educational Sciences	Director	Basic education	Master’s degree
G2	Male	45	Jiangsu Provincial Department of Education	Director	Physical education	Master’s degree
G3	Male	55	Jiangsu Provincial Sports Department	Director	Popular Sports	Master’s degree
G4	Male	56	Jiangsu Provincial Sports Department	Director	Adolescent sport	Master’s degree
G5^a^	Male	46	Lianyungang City Education Department	Deputy-director	Primary and secondary school education	Master’s degree

G = Government officer, a = Interview transcript for theoretical saturation test.

### 3.2 Open coding

The first step in theoretical sampling is open coding, which entails extracting initial concepts from all primary sources and editing and aggregating these concepts. This process requires the researcher to break down the raw data into actionable pieces for analysis, reflect on the data in memos, and summarise the data conceptually to form the initial concepts (Glaser et al., 1968). In this study, the initial ideas (A1–An) were created using Nvivo 12.0 to collate, categorise and summarise the interview recordings and notes, and coding them word by word to make the initial concepts. The initial ideas are refined, and the overlapping images are combined to form sub-categories. In the open coding process, 49 initial images (A1–A49) and 19 sub-categories (B1–B19) were obtained by refining representative statements. Table 5 shows the results of the open-ended coding, with information from some of the interviews listed in the table reflecting the open-ended coding process with representative initial statements.

**Table 5:** Results of open coding.

Initial concept (A)	Original text (representative sentence)
A1 Physiological value cognition	We’ve heard that playing basketball is suitable for growing taller, so we register for a basketball class for our son, and he also hopes to grow taller by playing basketball. (P8)
A2 Psychological value cognition	My boy’s idol is Federer (A tennis player), who wants to be a good tennis player like him. (P6)
A3 Social value cognition	The sports cultural festival held by our school is trendy among students. We all have our job and students say they could do some sports and make new friends at the festival. (T4)
A4 Hobby	Our kid has practised table tennis since childhood, and the critical point is that he likes the sport and would play it during holidays. (P5)
A5 Competitive struggle	Our school’s speciality is football. Our female team has gained the number one in the district, and they are ready to attend the municipal match, so they have a high passion for training. (H2)
A6 Sports confidence	My boy is on a PE Committee in the class. He has been in good health since childhood and has gained the Number 1 in the school sports meetings. (P1)
A7 Preference difference	Boys would like to move around, such as playing basketball or football; however, girls are different, and they would prefer to read or chat with each other in their free time. (T5)
A8 Exercise difference	My girl would feel okay with sports, but she would not attend some sports with strong antagonism (P4)
A9 Appearance and temperament	“Some boys are not that manly. They walk awkwardly without any male air, so how could they take activity? (H2)
A10 self-control	“Kids would play the game with IPAD in leisure time, and they are too excited to put it down.” (P8)
A11 Homework efficiency	“It only takes half an hour to finish homework if it is treated seriously, but the kid has to slow down to spend 1 h to finish it.” (P8)
A12 Understanding of sports	“Some parents say that playing football is time-wasting and nonsense” (T4)
A13 Cultural achievement orientation	“Parents still think highly of cultural achievement since PE is not the subject of the entrance examination to college” (T3)
A14 Overprotection	“Family sport is an important component of family education, but some parents overindulge children and worry them to get hurt from the sport so that they would prevent children from some sports with strong antagonism.” (G2)
A15 Parent’s company in doing sports	“Parents have strong working and family pressure; they are the backbone of the family, and they have no time to exercise with children or urge children to exercise, so most of the kids could not insist on the habit of doing exercise” (H2)
A16 Parent’s supervision in doing sports	“Sometimes a kid is so lazy, and we would supervise him and lecture him so that he could persist and never give up.” (P2)
A17 Parents’ role models in sports	In the family, parents are the role model of kids, so kids must love sports if their parents love them. Now many kids know of one or two sports skills since parents have cultivated them since their childhood consciously. This is very good! (T1)
A18 Kinship peer’s influence	The kid and his brother like skiing, and he often plays with him. (P8)
A19 Peer’s influence	“At the weekend or holiday, my kid would play basketball or swim with friends. They often go to play with several friends.” (P4)
A20 Economic income	“The economic status does not allow them to take part in extracurricular sports training.” (H1)
A21 Family composition	“Some kids grow up in a single-parent family or the grandparent’s family, so they lack parental care for a long time, and they would be weaker in both the physical or cultural achievement.” (H2)
A22 Site Equipment	“For example, there are many schools with a tiny campus in our township, and the old schools are hard to be reformed. The kids exercising must be affected due to the small venue, fewer resources and more students.” (G1)
A23 Safe and convenient passage	“School is not far away from home, and the transportation is good, as well. I would allow a kid to walk to school, and it often takes 15 min to get there.” (P2)
A24 Learning pressure	“The study in high school is so tight. The kid arrives at the school before 6:30 and goes home after 10 o’clock after finishing the self-study at night, so there is no time to do some sports.” (P3)
A25 Sports atmosphere	“Over half of students in the class positively attend the PE Cultural Festival, and the rest of them are willing to be a volunteer. The students like it so much.” (T4)
A26 Teacher resources	“There is only 1 PE teacher in the PE class of the junior high school, and the teacher is older, so what he could teach are some basic sports skills, such as rope skipping and running” (H2)
A27 Support from the principal	“Our leaders show great support, and they say that they would customise sports festival according to the feature of our school and there must be characteristics to be different from sports meeting” (T6)
A28 Support from the teacher	Teachers in school do think highly of their PE class. Mainly they are teachers of grade twelve, but teachers would often take some videos of them to organise students to have a running in school and upload them to the class group. (P1)
A29 Teaching methods of physical education	“Kid thinks the PE class is perfect since they could make a free selection and learn from different special teachers just like in college. You could attend the project you like instead of the fixed one.” (P1)
A30 Curriculum content of physical education	“There are many different sports items in our PE class, such as table tennis, badminton, and golf; added with figure exertion the weekend, there would be an extra swimming class. Our students like to have PE class so much.” (T4)
A31 Prevention of accidental injury	Since the present society values the accidental injury of students with much more attention, so the school would bear much more responsibility and pressure once there was something wrong. (H1)
A32 Equipment suitability	“Although there is fitness equipment around the residential quarter, they are not suitable for our kids since they are set for the aged people.” (P4)
A33 Equipment perfection	“Our residential quarter is old and broken. Unlike others, there is no fitness equipment.” (P8)
A34 Venue price	“Those stadiums are in a higher charge, so seldom students would spend money on doing exercise there.” (T1)
A35 Sports ground	There is no stadium around our residential quarter, so it would take a long time to find a place to do some exercise. (P3)
A36 Pedestrian greenways and bicycle lanes	“If there are much more bicycle lanes or pedestrian greenways, we would feel at ease when children ride or go to schools.” (P2)
A37 Public facilities for intelligent sports	“I have seen public facilities in foreign countries. To let children take fewer elevators and take more stairs, the stairs are designed as piano keys, and music will be played every time they walk. Such facilities can stimulate children’s interest and promote children to move” (T4)
A38 Perimeter security	“We won’t be assured when kid rids alone outside since there are much more social cars within the residential quarter” (P1)
A39 Residents’ complaints	“We would be complained by residents when playing basketball loudly in the downstairs in the weekend.” (P7)
A40 Extracurricular counselling class	“There are many more extracurricular counselling classes next to residential quarter, so I make a registration for the sake of my kid to keep learning well with others.” (P2)
A41 Community sports publicity	“Every year, teachers and students from the Institute of Physical Education come to us to promote health and guide fitness. The sports atmosphere is essential.” (P5)
A42 Impact of rain and snow weather	“We won’t allow kids to play outside on rainy and snowy days, and the PE class would be changed to be self-study class when it is rainy or snowy.” (P5)
A43 Impact of air quality	“It would be affected when running and doing exercise in the smog, and it would greatly affect students’ outdoor activity” (G1)
A44 Influence of temperature	“There used to be land for playing pickup. Now houses have been built, and there is no open space” (T1)
A45 Competition system	“In the past few years, the Ministry of Education’s “Campus Football League” and Jiangsu Province’s “Sunshine Sports League” has enabled much more students to take part in sports and exercise.” (G3)
A46 Evaluation system	“Our family, schools and society’s comment on children is mainly based on their cultural achievement, and the evaluation of selective test must be the comprehensive one, and there must be one for physical education. The current evaluation system could not show the importance of physical education.” (P6)
A47 Teaching system	“*Opinions on Deepening the Integration of Sports and Education and Promoting the Healthy Development of Teenagers* requires raising the baseline to ensure the teaching hours and ensure that there is 1 h for the campus and extracurricular physical activity every day.” (G3)
A48 Supervision and inspection	“The supervision of the Ministry of Education and the provincial and municipal supervisors would have a special patrol irregularly. It has not been a problem for our Jiangsu area to carry out sufficient PE class.” (G1)
A49 Venue blockade	“Before COVID-19, students could enter the basketball yard of school and workplace casually; however, they have been blocked during the period, and there is no place for activity” (H2)

### 3.3 Axial coding

The second central stage of coding is axial coding, which aims to discover and establish potential logical relationships between the subcategories and to combine the already refined subcategories relationally according to the principle of analogical relationships to form a more directed, selective and conceptual main category (Glaser et al., 1968). Our study used the socio-ecological model proposed by McLeroy et al. (1988) as a theoretical framework to generate 49 category connotations and 19 sub-categories in an open-ended coding process. Based on the results of the open-ended coding, we categorised the 19 sub-categories of the open-ended coding, resulting in five main categories (see Tables 6–10).

**Table 6:** Results of individual level axial coding.

SEM level	Main category	Subcategory	Category connotations
Individual level	Individual factors	B1 Self-cognition	A1 Physiological value cognition
			A2 Psychological value cognition
			A3 Social value cognition
		B2 Self-efficacy	A4 Hobby
			A5 Competitive struggle
			A6 Sports confidence
		B3 Personality and temperament	A7 Preference difference
			A8 Personality performance
			A9 Personal temperament
		B4 Time efficiency	A10 Self-control
			A11 Homework time

**Table 7:** Results of interpersonal level axial coding.

SEM level	Main category	Subcategory	Category connotations
Interpersonal level	Family environment	B5 Parent’s cognition of sports	A12 Understanding of sports
			A13 Focusing on cultural achievements
			A14 Overprotection
		B6 Support from parents	A15 Parent’s company in doing sports
			A16 Parent’s supervision in doing sports
			A17 Parents’ role models in sports
		B7 Family support	A18 Kinship peer
			A19 Peer’s influence
		B8 Household economy	A20 Economic income
		B9 Family atmosphere	A21 Family composition

**Table 8:** Results of organisation level axial coding.

SEM level	Main category	Subcategory	Category connotations
Organisation level	School environment	B10 School facilities	A22 Site Equipment
			A23 Safe and convenient passage
		B11 School atmosphere	A24 Learning pressure
			A25 Sports atmosphere
		B12 Teacher influence	A26 Teacher resources
			A27 Support from the principal
			A28 Support from the teacher
		B13 Teaching setting	A29 Teaching methods of physical education
			A30 Curriculum content of physical education
			A31 Prevention of accidental injury

**Table 9:** Results of community level axial coding.

SEM level	Main category	Subcategory	Category connotations
Community level	Community Environment	B14 Equipment configuration	A32 Equipment suitability
			A33 Equipment perfection
			A34 Venue price
			A35 Sports ground
			A36 Pedestrian greenways and bicycle lanes
			A37 Public facilities for intelligent sports
			A38 Perimeter security
		B15 Sports atmosphere in the community	A39 Residents’ complaints
			A40 Extracurricular counselling class
			A41 Community sports publicity
		B20 Natural factors	A42 Impact of rain and snow weather
			A43 Impact of air quality
			A44 Temperature impact

**Table 10:** Results of policy level axial coding.

SEM level	Main category	Subcategory	Category connotations
Policy level	Policy	B17 Teaching and Competition Policy	A45 Competition system
			A46 Evaluation system
			A47 Teaching system
		B18 Supervision and management policies	A51 Supervision and inspection
		B19 Epidemic control policy	A53 Venue blockade

#### 3.3.1 Individual level

At the individual level, interviewees discussed the factors that could have influenced physical activity among young people in terms of individual factors. Gender was the most talked about topic, with parents, teachers, principals and government officers discussing the differences in physical activity between boys and girls. Girls were less active than boys (some girls preferred to be sedentary, while boys preferred to move or play ball games). The differences in physical activity levels due to gender are consistent with the findings of previous quantitative studies, which tend to be the result of pre-deposited biological factors. In addition, most parents interviewed felt that their children were physically active enough. Some parents and teachers responded that they were not satisfied with their children’s use of time as a cause of lack of time for physical activity, given their children’s preference to use electronic devices in their free time and lower efficiency in doing homework.

#### 3.3.2 Interpersonal level

At the interpersonal level, all respondents indicated that the influence of the family environment significantly influenced children’s physical activity. Parental support was the most discussed topic in the family environment and was seen as the primary facilitator of children’s participation in physical activity. Parents were role models for their children; if they maintained an active lifestyle, their children would also become more active. In addition, parental sports accompaniment and sports supervision were all effective means of promoting physical activity in children.

Some teachers interviewed reported that some parents had a low awareness of PE and a bias towards the subject. They believed PE was not as important as the main subjects and did not bring benefits to the Chinese College Entrance Examination (CCEE). Some parents used ‘no sports time’ as a punishment and forbade their children to participate in physical activity if they did not achieve the desired academic performance. So, parents’ low awareness of the benefits of sports participation can prevent children from participating in physical activity.

Some government officers argued that ‘over-protection’ by parents could also discourage young people from participating in physical activity, as they were overly concerned about their children getting injured in sports and prohibited their children from participating in some highly competitive sports.

Regarding the family atmosphere and finances, some teachers said that children from single-parent families and families with more complex financial situations showed poorer performance in sports and did not participate in sports in general. Children from single-parent or intergenerational families might be more difficult having their parents or guardians accompany and supervise them and to develop exercise habits. The same is true for children from economically challenged families, where family income could limit spending on sports.

Some principals and teachers reported that in children with siblings, especially those of similar age, their physical activity levels were usually higher, and the siblings became more active together. Similarly, parents gave feedback that when children were with their peers, they became more active and often met up with friends to play sports such as football.

##### 3.3.2.1 Organisation level

At the organisation level of social ecology, the school is the most critical location for adolescents to engage in physical activity. Adolescents spend most of their day at school, and the school environment directly impacts adolescent physical activity (Hou and Liu, 2019). This was confirmed in the research of this chapter, where teachers, principals, and government officers interviewed indicated that physical education facilities in the school environment had a direct impact on adolescents’ physical activity levels, with factors such as sports facilities, activity spaces, and the size of the residential district where the school is located, all affecting physical activity levels. Some parents reported that if the school was well-connected and not too far from home, their children could walk daily to and from school. Therefore, the accessibility and safety of the area around the school also contributed to the children’s physical activity level.

Parents and teachers generally believed that excessive academic pressure prevented young people from engaging in adequate levels of physical activity. The heavy academic load could lead to students being sedentary for long periods and not having enough time to participate in physical activity. Government officers had also stated that this was a common problem in schools today and that there were adverse health consequences for young people who were not physically active during the school days. However, some principals said that academic pressure and physical activity were not incompatible, as academic stress could not be avoided as children had to face secondary and higher education exams shortly. Physical activity is equally important and can be promoted in other ways, such as increasing the number of physical education hours, adjusting the content of physical education classes, and offering physical culture festivals.

Five out of nine parents indicated that the support and assistance of teachers were essential in promoting physical activity among young people. Parents reported that teachers’ supervision and the requirement to check off physical activity assignments in the social media groups, such as QQ and WeChat groups, had improved physical activity levels among young people.

Three out of seven teachers interviewed said that with the support and decision-making of school leaders, it would be much smoother for the campus to conduct sports competitions and sports culture festivals, and that the principal’s concern for sports and sports philosophy could directly drive the sports atmosphere across the campus. In addition, some teachers believed there was a severe shortage of PE teachers in schools and that they were generally too old to keep up with the times in terms of teaching methods, which could affect students’ learning of PE skills.

The richness of the PE curriculum and the variety of teaching methods could affect the students’ perception of psychological value, which indirectly affected physical activity levels. For example, boring running, rope skipping, or specific exercises for PE mid-term exams could make students feel bored and unmotivated, whereas ball games and aerobics were more popular among students. During the interviews, we learned that some schools had adopted an option-based and club-based approach to delivering PE lessons, providing students with various options to stimulate their interest in participating in physical activity. Some teachers interviewed said that the intensity of PE lessons could be reduced for safety reasons. However, on the other side, the reduced intensity and confrontation of PE lessons might result in students not achieving the required amount of exercise in class.

##### 3.3.2.2 Community level

According to the parents interviewed, the lack of fitness equipment, gymnasiums, and sports venues in the communities where they lived had a negative impact on the daily physical activity of young people, and the fact that high costs for using sports venues and gymnasiums in the neighbourhood were also a deterrent for ordinary families. Parents believed building walking and cycling paths as part of urban planning or neighbourhood renewal would be a good idea. The construction of walking and cycling paths would positively impact young people’s physical activity.

Parents reported that “the sporting atmosphere in the community has a direct impact on the physical activity of young people, sometimes children play in the basketball court in the neighbourhood but are often being complained about by older residents who like to keep quiet,” “there are various sports training institutions around the neighbourhood, so parents also enrol their children in training courses in the neighbourhood,” “the fees are too expensive,” and “the fees are too high.” “Posters on exercise and health are often displayed in the community; now and then, community workers come to the neighbourhood to promote scientific fitness methods and free blood pressure and blood sugar tests.” These factors could directly or indirectly influence young people’s physical activity levels.

In addition, parents and teachers believed that weather conditions could also have a significant impact on physical activity when the children were out of school. Especially when it came to rain, snow, or hazy weather, the degree of warm and cold weather also affected their physical activity to some extent.

##### 3.3.2.3 Policy level

At the policy level, the school principals interviewed felt that the school sports events organised in recent years had motivated students and promoted physical activity among young people, such as the National School Football League organised by the Ministry of Education, which requires junior and senior secondary schools to participate in football training classes and the schools to participate in the national tournament after a competitive selection process. The abundance of competitions and the in-school selection system contributed to young people’s physical activity.

The parents interviewed said that the current education evaluation system was biased and that the value and significance of PE were not reflected in the educational performance evaluation system, especially in the recent selection tests, where the percentage of PE credits in the secondary school exams was not adequate, and the difference in PE achievements between students was not significant; the high school exams did not include PE scores, and PE classes were reduced to optional. The bias of the current education evaluation system was the root cause of the “emphasis on literature rather than martial arts” phenomenon, which was directly responsible for the low level of physical activity among young people. In addition, the lack of clear rewards and sanctions for PE in schools also affected students’ motivation to participate in activities. The current evaluation system was considered to directly impact young people’s physical activity levels.

Teachers, principals and government officers agreed that a series of physical education reform policy documents had contributed to young people’s physical activity in recent years. While previous policy require 1 h of daily physical activity for students, current documents required 1 h in and 1 h out of the school time. In addition, some principals suggested that implementing the “double reduction” policy (It refers to a policy implemented in China’s education system aimed at reducing the workload of primary and junior high school students in terms of homework and extracurricular training) had significantly reduced students’ school workload, leaving more time for leisure and physical activity. As a result, the policy from the education system directly impacted young people’s physical activity.

The teachers and government officers interviewed felt that the rigorous school monitoring process in Jiangsu Province had ensured that young people were physically active and that the provision of adequate physical education was no longer an issue in Jiangsu, but that the goal for the future was to establish a social monitoring mechanism and that a ‘two-pronged’ inspection policy would promote physical activity among young people.

Finally, some principals said that the (COVID-19) epidemic prevention and control policy could lead to a lack of physical activity among young people, as the closure of schools and public sports venues could have affected their physical activity.

#### 3.3.3 Selective coding

After the open-ended and axial coding, selective coding is the last step in grounded theory analysis, where researchers select a core category and connect all the other categories around that core category. This process results in one unified theory that explains the research findings. Selective coding is a process that aims to extract the essence of the idea, which can be closely linked to other conceptual types to form a generalised theory (Glaser et al., 1968). The goal of selective coding is to extract the core categories from the main categories; and once the core categories were identified, they were linked to other classes through the model, and the links between the types had been hypothesised in the literature (Glaser et al., 1968; Mik-Meyer, 2020).

In the selective coding stage, this study repeatedly compared and analysed in depth the five main categories (individual factors, family environment, school environment, community environment and policy), formed in the axial coding stage, combined with the careful collation of the original interview data, systematically sorted out the relationships between the main categories and the factors influencing adolescents’ physical activity, and finally, after categorisation and refinement, formed 10 relationship structures, as shown in Table 11.

**Table 11:** Selective coding.

Inter-category relations	Relationship structure	Representational statements
Individual factors → PA	Causal relationship	“The child goes to play basketball whenever he can because he likes it” (P7)
Family environment → Individual factors	Intermediary relationship	“There are times when my son is lazy and does not want to exercise, so we encourage him and tell him to persevere and not to give up” (P2)
School environment → Physical activity	Causal relationship	“Every time the school has a sports and cultural festival, more than half of the people in each class volunteer to participate in the activities” (T4)
School environment → individual factors	Intermediary relationship	“The teachers at school still pay much attention to their PE lessons, especially now that the children are in their senior year; the teachers often urge them to get active and will often make exercise videos for them to follow to relieve stress (P1).”
School environment → Family environment	Intermediary relationship	“Because of the PE mid-term exams, we ask parents to monitor their children’s exercise at home and clock in at least three jump ropes and three standing long jump jumps per week” (T4)
Community environment → PA	Causal relationship	“If there were more bike lanes or walking greenways around the neighbourhood, then we would be relieved when our child rides his bike or let him walk to and from school. (P2)
Community environment → individual factors	Intermediary relationship	“We have fitness equipment in the community, but the children do not like it; it’s some equipment used by older people” (P4)
Community environment → Family environment	Intermediary relationship	“Our community has a soft track, tennis courts, and a swimming pool, and I do quite like to take my son with me to play sports” (P6)
Policy → PA	Causal relationship	“The Ministry of Education’s policy calls for an increase in the bottom line of physical education hours in schools, with one hour of physical activity each in and out of school each day” (G2)
Policy → School environment	Intermediary relationship	“Since the launch of the ‘School Football’ campaign, there are now 2,000 schools featuring school football in our Jiangsu region.” (G3)

PA = Physical activity; P = parents; T = teachers; G = government officers.

**Figure 2:**
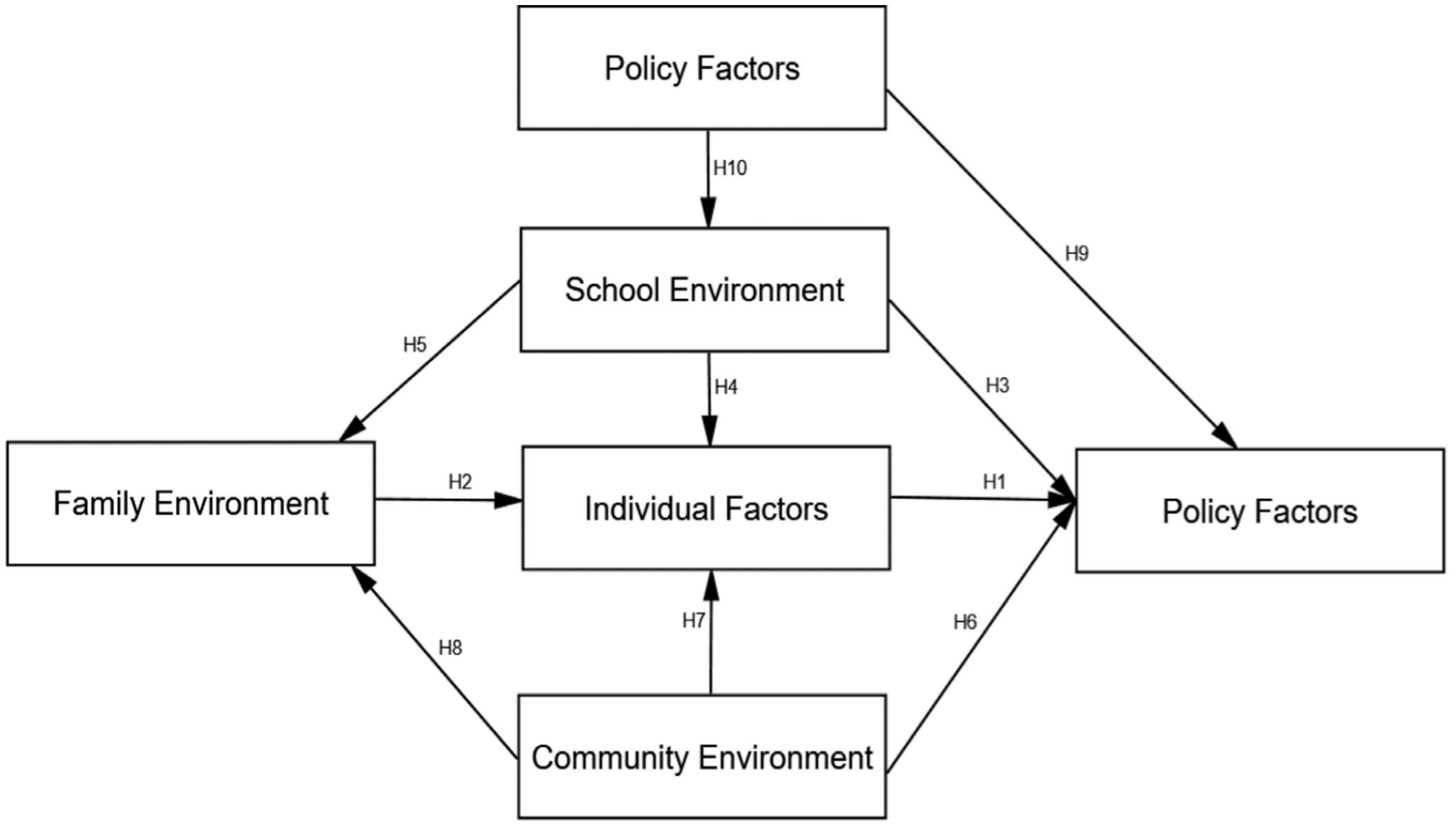
Initial conceptual model of factors influencing physical activity in adolescents. H = hypothesis.

By analysing the linkages between the categories, the core categories that dominated the other major categories were extracted, and a conceptual model was constructed using an ‘unfolding storyline’ approach to describe the overall behavioural phenomena and contextual conditions (Glaser et al., 1968). The final core categories were: individual factors, family environment, school environment, community environment, and policy, and were developed through two storylines: “core category → youth physical activity” and “core category interaction → youth physical activity.” The initial model of the factors influencing adolescent physical activity was finally constructed, as shown in Figure 2.

#### 3.3.4 Research hypotheses

Direct and indirect effects describe the relationships among observed and latent variables in the model (Charmaz and Thornberg, 2021; Oktay, 2012). Direct effects refer to the direct influence of one variable on another, while indirect effects refer to the indirect influence of one variable on another through one or more intermediate variables. Figure 2 shows two pathway relationships of influencing factors: (1) Direct influence: individual factors, school environment, community environment, and policy all directly influence adolescent physical activity levels; (2) Indirect influence: community environment indirectly influences youth physical activity through the family environment and unique environment; policy indirectly influences youth physical activity through the school environment, family environment, and individual factors. Specifically, the following research hypotheses were formed.

H1: Individual factors → physical activity has a positive influence.

H2: The family environment has a positive impact on individual factors.

H3: The school environment has a positive effect on physical activity.

H4: School environment → individual factors have a positive effect.

H5: School environment → family environment has a positive influence.

H6: Community environment → physical activity has a positive effect.

H7: Community environment → individual factors have a positive effect.

H8: Community environment → family environment has a positive influence.

H9: Policy → physical activity has a positive effect.

H10: There is a positive effect of policy → school environment.

#### 3.3.5 Model construction and analysis

In this study, through the semi-structured interviews with parents, teachers, principals and government officers, and using the social ecology model as the theoretical framework, the researcher coded and analysed the interview texts and concluded that the main factors affecting youth physical activity included: individual factors, family environment, school environment, community environment and policy. This led to the construction of the Initial Model of Influencing Factors on Youth Physical Activity (Figure 2), which was structured with the following critical conceptual relationships.

1. Young people’s participation in physical activity was influenced by multiple factors, and the interrelationships were complex.
2. Individual factors included self-perception, self-efficacy, personality, and time efficiency, which were internal situational conditions that influenced adolescents’ physical activity and positively impacted physical activity behaviour.
3. The family environment included five dimensions: parental support, parental perception, family support, family economy, and family atmosphere, which were external situational conditions that influenced adolescents’ physical activity.
4. The school environment included facilities, climate, teachers’ influence, and curriculum. These dimensions were external situational conditions that influenced adolescents’ physical activity. The school environment could either directly and positively impact adolescents’ physical activity levels or indirectly influence physical activity levels through mediating variables.
5. The community environment included facilities, community sporting atmosphere, and natural factors, which were external situational conditions affecting youth physical activity.
6. Policy factors included teaching and competition policies, supervision and management policies, and epidemic prevention and control policies, which could directly and positively influence youth physical activity levels and indirectly influence physical activity levels through mediating variables.
7. Individual factors were the core category of factors influencing youth physical activity; family environment, school environment, and community environment were the keys to participation in physical activity; and policy factors were the guarantee of youth physical activity.

#### 3.3.6 Theoretical saturation test

This study followed the principle of theoretical saturation. The purpose of the theoretical saturation test was to verify that the physical activity factors studied for adolescents were comprehensive and that the data collected covered all aspects of the respondents’ concepts, content, and connotations. The theoretical saturation test assesses the adequacy of the coding in the academic sampling process. If new ideas or definitions are identified during the trial, indicating that the theory is not saturated, further additional information needs to be collected to supplement the data. Based on the concepts defined by the open-ended coding, the categories formed, and the naming of the classes for a continuous round-robin examination to ensure the scientific validity of the refinement from concepts to types, we re-coded the remaining one parent interview transcript (P9), one teacher interview transcript (T7), one principal interview transcript (H4) and one government official interview transcript (G5) for analysis according to the above procedure, respectively, in that the coding process did not produce new conceptual connotations or new causal relationships, so it can be argued that the richness of the categories covered in this grounded theoretical model had reached saturation.

## 4 Discussion

In this study, we examined the factors that could influence the physical activity of our youth at five levels using the social ecology model proposed by McLeroy et al. (1988) as a theoretical framework.

### 4.1 Individual factors

The study’s results suggest that the personal factors influencing physical activity include self-perception, self-efficacy, personality temperament, and time efficiency. The path of influence in the model was: individual factors → adolescent physical activity, indicating that adolescent physical activity is directly influenced by individual factors and that individual factors were internally causally related to adolescent physical activity. Consistent with previous findings, scholars have previously suggested that adolescent physical activity levels are directly related to individual factors such as psychological, physiological, and time management (Bengoechea et al., 2013; Stanley et al., 2013).

Psychologically, the levels of self-perception and self-efficacy significantly and positively affected physical activity levels, as found in this study. Self-perception, which indicates an individual’s insight and understanding of themselves, correlates statistically with physical activity levels (Ge et al., 2015; Zhang et al., 2012). “Self-efficacy,” a concept developed by Bandura in the 1970s, refers to an individual’s confidence in their ability to use what they have to perform a behaviour (Bandura, 1977). It has been suggested that people tend to choose actions or tasks that they feel interested in or confident in completing. When individuals have a high level of “self-efficacy,” they are motivated to engage in the task or activity (Zhang et al., 2012). “Hobby,” “competitive striving,” and “athletic confidence” are the same categories in which “self-efficacy” is embedded.

Physiologically, it has been suggested that personality traits are a key factor in adolescent participation in physical activity (Guo and Wang, 2020) and that there are differences in physical activity between boys and girls, both in terms of amount and type of physical activity (Pawlowski et al., 2014). Our interviews also reflected this view, where parents and teachers generally responded that girls preferred a sedentary lifestyle. In contrast, boys preferred more confrontational physical activities, such as various ball sports events. In this study, we found that individual factors such as ‘temperament’ and ‘time efficiency’ had an impact on physical activity participation behaviour, which has rarely been discussed in previous studies.

### 4.2 Family environment

Our findings suggested that the family environment influenced physical activity in five dimensions: parental support, parental perceptions, family support, family finances and family climate. The path of influence in the model was: family environment → individual factors, → adolescent physical activity, indicating that the family environment indirectly influenced adolescent physical activity levels by using individual factors as mediating variables. Our findings are consistent with previous studies, in which Kahn et al. (2008) and Liu et al. (2021) concluded that support from parents and friends could enhance children and adolescents’ self-efficacy and self-perceptions, thus indirectly contributing to physical activity levels. At the same time, our study suggests that the family environment has a direct and positive effect on individual factors in adolescents. Parental support,” “parental perception” and “family atmosphere” in the family environment have an immediate positive impact on the individual factors of children’s “self-perception” and “self-efficacy.” “The family environment, including ‘parental support’, ‘parental perceptions’ and ‘family atmosphere, can have a significant impact on individual factors such as children’s ‘self-perceptions’, ‘self-efficacy’, ‘personality traits’ and ‘time efficiency. The interviews revealed that parental supervision enhanced children’s time management efficiency; parental exercise habits developed children’s perception of sports; parental encouragement and support enhanced children’s self-efficacy, and a harmonious family atmosphere developed children’s temperament and personality. The results of our study are in line with Dagkas and Quarmby (2012) and Huang et al. (2019), which concluded that the family environment directly impacts individual factors of adolescents.

### 4.3 School environment

The results of this study suggested that the school environment influenced physical activity in four dimensions: school facilities, climate, teacher influence, and curriculum. The pathways of power in the initial model suggest that the school environment can influence physical activity levels both directly and indirectly through the home environment and individual factors as mediating variables. Schools are considered the best place to practise wellness programmes and implement physical activity interventions (Hou and Liu, 2019). Some scholars have discussed the influence of the school environment on adolescents’ physical activity levels in terms of school facilities, physical education climate, and teacher influence and concluded that the school environment has significant predictive power for physical activity behaviour in adolescents (Chen et al., 2018; Dai and Chen, 2019; Hou and Liu, 2019). Some scholars have also indicated that increasing the number of physical education classes by adjusting the delivery of school physical education courses is an effective way to promote physical activity among adolescents (Luo et al., 2021; Pawlowski et al., 2014; Stanley et al., 2012). The results of previous studies have shown the importance of the school environment on physical activity, and we have come to the conclusion, consistent with our predecessors, that physical activity can be directly and positively promoted among adolescents in schools by improving school physical education facilities, spreading school physical education culture, optimising the content of physical education courses and enhancing physical education teachers.

### 4.4 Community environment

The results of this study showed that the community environment influenced physical activity in three dimensions: facility configuration, community sporting atmosphere and natural factors. The influence pathway in the initial model showed that the community environment could influence physical activity levels both directly and indirectly through family environment and individual factors as mediating variables. Regarding the influence of the community environment, our results are consistent with previous studies (D'Angelo et al., 2017; Yan et al., 2014) that describe the impact of community sports facilities and community sporting climate on youth physical activity. For example, lack of fitness equipment and sports fields, and lack of walking greenways and bicycle paths can constrain youth physical activity participation (Wilk et al., 2017). Therefore, improving accessibility, promoting active transportation and enhancing community sports facilities are helpful in increasing youth physical activity levels (D'Angelo et al., 2017; Luo et al., 2021; Stanley et al., 2012). In addition, the current study found that environmental factors such as climatic conditions and air quality could also affect youths’ physical activity participation, in line with studies by scholars such as Li et al. (2021) and Huang et al. (2019).

### 4.5 Policy

The results of this study suggested that policy factors affecting physical activity included three dimensions: teaching and competition policy, supervision and management policy, and epidemic prevention and control policy. Previous studies on youth physical activity have rarely addressed policy-level factors, probably because the venues for youth physical activity are mainly in the community or schools, so most previous studies have focused on schools and communities for investigation. In this study, in order to provide a more comprehensive overview of the factors influencing adolescent physical activity, and also to quantify the effect of policy on physical activity, we used the McLeroy et al. (1988)’s social ecology model as a theoretical framework to summarise the policy factors using rooting theory and proposed a pathway for the influence of policy factors on physical activity. The pathways of influence in the initial model showed that policy could affect physical activity levels both directly and indirectly through the school environment as a mediating variable. Our findings are consistent with foreign scholars, as Langille and Rodgers (2010) stated that “physical education policies or health promotion policies developed at the provincial (state) and municipal levels have a top-down, one-way path of influence on schools.” The implementation of the policy relies on the school as a platform and therefore the school environment plays a mediating role in the implementation of the policy.

From the initial conceptual model we can see that individual factors, as a central category in adolescent physical activity, have a direct impact on physical activity behaviour, but that individual factors are themselves subject to influence from the external environment. Bandura argues that “when environmental conditions exert a powerful influence on individual behaviour, they become the overriding determinant (Bandura, 1986). The family environment, school environment and community environment play a key role in adolescent physical activity as extra-individual environmental factors, either directly or indirectly through individual factors. Therefore, the integration of the family, school and community environments is extremely important in promoting physical activity among young people. As one government official (G1) stated, “Promoting physical activity among young people requires the cooperation of home, school and community.” Schools provide the physical education curriculum, while the home and community environment is a useful extension and supplement to the school physical education curriculum. In future, the promotion of youth physical activity will require a shift from the school-led approach to a ‘trinity’ of family, school and community partnerships to effectively improve youth physical activity levels. The policy factor is the outermost and most macroscopic factor in the social ecology model proposed by McLeroy et al. (1988) and its impact on physical activity is most extensive. In recent years, the Chinese government has promulgated various policy documents to support this in order to improve the level of physical activity and promote the physical health of young people, for example, in August 2020, the *Opinions on Deepening the Integration of Physical Education and Promoting the Healthy Development of Youth* was jointly issued by the Ministry of Education and the General Administration of Sports of China, and in October of the same year, the General Office of the Central Committee of the Communist Party of China and the State Council issued the *Opinions on comprehensively strengthening and improving school physical education work in the new era*. These policy documents provide ideas and bases for the reform of school sports development. In the process of implementing the policies, it is necessary to develop a monitoring and inspection system to prevent formalism and laissez-faire in order to guarantee the implementation of physical exercise standards for young people and to ensure that students have 1 h of physical activity both within and outside school during school days.

## 5 Limitations

Despite the valuable insights generated, this study has several limitations. First, the sample size of 25 participants, while sufficient for data saturation in qualitative research, may limit the generalizability of the findings. Second, the study was conducted in Jiangsu Province, and the findings may not fully represent experiences in other regions of China. Future research should address these limitations by incorporating larger samples, including adolescents’ perspectives, and expanding to different geographical areas.

## 6 Conclusion

This study utilised semi-structured interviews with teachers, principals, government officers, and parents to assess the factors influencing adolescent physical activity in China. Utilising the grounded theory within the social ecology model framework, the research identified 49 concepts across 19 subcategories and five main categories. The resulting initial model, which integrated the five main categories, provided a foundational understanding of the multifaceted influencing factors of adolescent physical activity in China.

To further promote adolescent physical activity, policies should focus on strengthening physical education programmes and encouraging collaboration between families, schools, and communities, while future research should include adolescents’ perspectives and explore regional differences and long-term policy impacts.

## Data Availability

The raw data supporting the conclusions of this article will be made available by the authors without undue reservation.
